# Comparative Transcriptomic and Metagenomic Analyses of Influenza Virus-Infected Nasal Epithelial Cells From Multiple Individuals Reveal Specific Nasal-Initiated Signatures

**DOI:** 10.3389/fmicb.2018.02685

**Published:** 2018-11-14

**Authors:** Kai Sen Tan, Yan Yan, Wai Ling Hiromi Koh, Liang Li, Hyungwon Choi, Thai Tran, Richard Sugrue, De Yun Wang, Vincent T. Chow

**Affiliations:** ^1^Department of Otolaryngology, National University of Singapore, Singapore, Singapore; ^2^Center for Interventional Medicine, The Fifth Affiliated Hospital of Sun Yat-sen University, Zhuhai, China; ^3^Saw Swee Hock School of Public Health, National University of Singapore, Singapore, Singapore; ^4^Institute of Biomedicine and Biotechnology, Shenzhen Institutes of Advanced Technology, Chinese Academy of Sciences, Shenzhen, China; ^5^Institute of Molecular and Cell Biology, A^∗^STAR, Singapore, Singapore; ^6^Department of Physiology, National University of Singapore, Singapore, Singapore; ^7^School of Biological Sciences, Nanyang Technological University, Singapore, Singapore; ^8^Department of Microbiology and Immunology, Yong Loo Lin School of Medicine, National University of Singapore, Singapore, Singapore

**Keywords:** influenza, transcriptomics, meta-analysis, human nasal epithelial cells, pre-clinical model

## Abstract

*In vitro* and *in vivo* research based on cell lines and animals are likely to be insufficient in elucidating authentic biological and physiological phenomena mimicking human systems, especially for generating pre-clinical data on targets and biomarkers. There is an obvious need for a model that can further bridge the gap in translating pre-clinical findings into clinical applications. We have previously generated a model of *in vitro* differentiated human nasal epithelial cells (hNECs) which elucidated the nasal-initiated repertoire of immune responses against respiratory viruses such as influenza A virus and rhinovirus. To assess their clinical utility, we performed a microarray analysis of influenza virus-infected hNECs to elucidate nasal epithelial-initiated responses. This was followed by a metagenomic analysis which revealed transcriptomic changes comparable with clinical influenza datasets. The primary target of influenza infection was observed to be the initiator of innate and adaptive immune genes, leaning toward type-1 inflammatory activation. In addition, the model also elucidated a down-regulation of metabolic processes specific to the nasal epithelium, and not present in other models. Furthermore, the hNEC model detected all 11 gene signatures unique to influenza infection identified from a previous study, thus supporting the utility of nasal-based diagnosis in clinical settings. In conclusion, this study highlights that hNECs can serve as a model for nasal-based clinical translational studies and diagnosis to unravel nasal epithelial responses to influenza in the population, and as a means to identify novel molecular diagnostic markers of severity.

## Introduction

Annually, influenza virus infection causes a large number of vaccine-preventable deaths worldwide (Poland et al., 2007). Despite global efforts to curb ongoing transmission, novel strains of influenza viruses are emerging at a rapid rate, and threaten to escalate into epidemics or pandemics (Hui et al., 2017). Therefore, there is an urgent need to enhance our understanding of influenza pathogenesis to discover means to ameliorate the global burden of influenza virus infection.

The nasal epithelium is the primary portal of entry for respiratory viruses such as influenza viruses, and serves as an immediate target for viral replication in the respiratory tract (Braciale et al., 2012; Kolesnikova et al., 2013; Yan et al., 2013, 2016; Wang D.Y. et al., 2015). The nasal epithelium was initially thought to function as a mechanical barrier that filters external agents, preventing their entry into the respiratory system; but recent studies have revealed that it functions beyond just being a physical barrier (Watelet et al., 2006; Wang D.Y. et al., 2015). Nasal epithelial cells are shown to elicit their own repertoire of immune responses and actively prevent pathogens from damaging the airway (Yan et al., 2013, 2016). Upon infection, they not only release anti-microbial surfactants and mucus to mitigate pathogen transmission in the airway (Watelet et al., 2006; Wang D.Y. et al., 2015), but also express and secrete various cytokines and chemokines to drive immune responses against these pathogens (Watelet et al., 2006; Yan et al., 2016). Therefore, the interactions between the nasal epithelium and invading pathogens play key roles in the disease progression and subsequent immune responses against the virus.

Despite direct interaction with the virus at host contact sites, studies on influenza viruses often overlook the nasal epithelia, but focus on the lungs where the more serious influenza-induced pneumonitis occurs. As a result, there is a lack of suitable nasal epithelial models in the current literature (Sutejo et al., 2012; Watanabe et al., 2013; Clay et al., 2014; Wu et al., 2014; Wang Z. et al., 2015). Therefore, it is of interest to profile nasal epithelial responses to influenza virus at the actual contact site, and to characterize the nasal epithelium as an intermediate checkpoint for downstream immune responses by immune cells (e.g., secretome). Furthermore, it is important to determine whether molecular factors that confer differential susceptibility among individuals with different genetic backgrounds exist in the nasal epithelium, thereby impacting influenza transmission in the host population. The investigation of nasal-based markers and susceptibility factors may also encourage nasal-based diagnosis that can be developed into a rapid tool for influenza management.

To investigate the feasibility of nasal models in elucidating influenza signatures, we have developed an *in vitro* model of fully differentiated, multi-layered nasal epithelial cells derived from stem cells of different individuals in an air-liquid interface (ALI) culture (Li et al., 2014; Yan et al., 2016). This model fully mimics nasal epithelium with the presence of ciliated, goblet and basal cells. The model also facilitates focused analysis of epithelial responses, and their contribution to anti-influenza responses (Yan et al., 2016). Using this *in vitro* ALI model, we studied the transcriptomic signature of the nasal epithelium in response to influenza virus infection. We hypothesized that the *in vitro* differentiated nasal epithelium closely mimics immune responses in cell lines and blood samples from clinical studies, and serves as the initial checkpoint of these responses. We compared the infected nasal epithelial transcriptomic signature against influenza infection signatures derived from other studies that employ epithelial cell lines or patient blood samples through meta-analysis to highlight responses triggered by the nasal epithelium. The objective of this study is to evaluate the utility of the hNEC model as a bridge between pre-clinical data and clinical settings, in order to achieve its potential application in clinical influenza and infectious diseases.

## Materials and Methods

### Derivation of hNESPCs and *in vitro* Differentiation of hNECs

This study was approved by the Ethical Committees (National Healthcare Group of Singapore Domain-Specific Review Board, DSRB Reference no. D/11/228, and National University of Singapore Institutional Review Board, IRB code 13-509). Under the DSRB and IRB, written informed consent was obtained from each subject. The human nasal epithelial stem and progenitor cells (hNESPCs) were derived from the inferior turbinate of five patients with septal deviation (SD) who underwent septal plastic surgery at the National University Hospital, Singapore. All subjects were free of symptoms of upper respiratory infection, and had not used corticosteroids and antibiotics within 3 months before the surgery. The medical background of the donors’ samples is summarized in Supplementary Table S1. Cell culture method has been described previously (Zhao et al., 2012; Li et al., 2014; Wang et al., 2014). Fully differentiated hNECs, including beating ciliated cells and mucus-producing goblet cells, were obtained after 32–35 days of ALI culture. The hNECs were previously characterized by immunofluorescence (IF) staining of ciliated and goblet cell markers, i.e., mouse anti-human βIV-tubulin (Zhao et al., 2012; Li et al., 2014), rabbit anti-human acetylated α-tubulin and rabbit anti-human mucin5AC (Wang et al., 2014).

### Influenza A Virus (H3N2) Infection of Fully Differentiated hNECs

The human influenza A virus Aichi/2/1968 H3N2 strain was purchased from the American Type Culture Collection (ATCC, Manassas, VA, United States), propagated in eggs, and titrated by plaque assay using Madin-Darby canine kidney (MDCK, NBL-2) cells (ATCC, Manassas, VA, United States). The virus was thawed on ice and immediately diluted in 100 μL of B-ALI^TM^ differentiation medium at a multiplicity of infection (MOI) of 0.1, inoculated into the apical chamber of Transwells, and incubated at 35°C for 1 h. B-ALI^TM^ differentiation medium was added into the control well at 0 h post-infection (hpi) without the virus. The H3N2-infected and mock-infected hNECs were then incubated at 35°C with 5% CO_2_ for 8, 24, and 48 hpi, respectively.

### Virus Plaque Assay

At serial time-points, 150 μL of DPBS was added and incubated in the apical chamber at 35°C for 10 min to recover progeny viruses, and the aliquots of inoculated virus were stored at −80°C until titration by plaque assay. MDCK cells at 85–95% confluence in 24-well plates were incubated with 100 μL of serial dilutions (from 10^−1^ to 10^−4^) of virus from infected hNECs at 35°C for 1 h. The plates were rocked every 15 min to ensure equal distribution of virus. The inocula were removed and replaced with 1 mL of Avicel overlay (FMC Biopolymer, Philadelphia, PA, United States) to each well, and incubated at 35°C with 5% CO_2_ for 65–72 h. Avicel overlay was then removed, and cells were fixed with 4% formaldehyde in PBS for 1 h. Formaldehyde was removed, and cells were washed with PBS. The fixed cells were stained with 1% crystal violet for 15 min, and washed. The plaque-forming units (PFU) were calculated as follows: number of plaques × dilution factor = number of PFU per 100 μL. The final data were presented as PFU per 100 μL (innoculation volume).

### Total RNA Extraction and Gene Microarray

Total RNA was extracted from hNECs using mirVana^TM^ miRNA isolation kit (Life Technologies, Carlsbad, CA, United States) following the manufacturer’s protocol. The concentration and quality of total RNA were determined by a bio-analyzer, and only total RNA samples deemed of good quality were subjected to microarray analyses. Gene microarrays for the infected and control hNEC samples were performed using Affymetrix human transcriptome array (HTA) microarray platform.

### Reverse Transcription and Real-Time Quantitative PCR

RNA (2 μg) was subjected to cDNA synthesis using the Maxima first-strand cDNA synthesis kit (ThermoScientific, Pittsburgh, PA, United States). The qPCR analysis was performed to evaluate the transcriptional levels of host response genes selected based on the microarray analysis using pre-designed primers (Sigma Aldrich, St. Louis, MO, United States). The qPCR assays were performed in duplicate using GoTaq-qPCR Master Mix kit (Promega, San Luis Obispo, CA, United States), and the median values and interquartile ranges were generated. Relative gene expression was calculated using the comparative method of 2^−ΔΔCt^, i.e., 2^(ΔCtofgene^^−^^ΔCtofPGK1)^, and normalized to the mRNA level of the housekeeping gene PGK1.

### Bioinformatics Analysis for Gene Expression Data in hNEC Samples

Affymetrix microarray data for hNEC samples were processed using the “affy” package in R Bioconductor (Gautier et al., 2004). A Bayesian model-based method was to detect differential expression between post-infection time-points and baseline (Teo et al., 2015). A gene was considered differentially expressed if the probability score for differential expression was above the threshold associated with 1% FDR. Pathway-level analysis was conducted in two ways, using the Ingenuity Pathway Analysis (IPA; Qiagen, Hilden, Germany) and an in-house program for testing enrichment of biological functions (hypergeometric tests) against a database consisting of Gene Ontology (Ashburner et al., 2000) and CPDB (Kamburov et al., 2009).

### Metagenomics Analysis of hNEC Transcriptome With Multiple Influenza Transcriptomic Studies

The studies used in the meta-analysis are summarized in Supplementary Table S2. All processed data sets were downloaded from the Gene Expression Omnibus (GEO) database after literature search. The same model-based differential expression analysis was used for comparison between infection peak time and the baseline. For each and every data set, systematic shifts in the expression values were examined by boxplots and it was deemed unnecessary to apply further normalization procedures. The list of up- and down-regulated genes was also annotated in terms of biological functions in each study, and compared across the studies along with the hNEC data (−log10 *p*-value for up-regulation and log10 *p*-value for down-regulation to account for direction of change).

## Results

### The hNECs of Multiple Subjects Reveal Similar Temporal Responses Against Influenza Infection With Varying Magnitude of Expression

**FIGURE 1:**
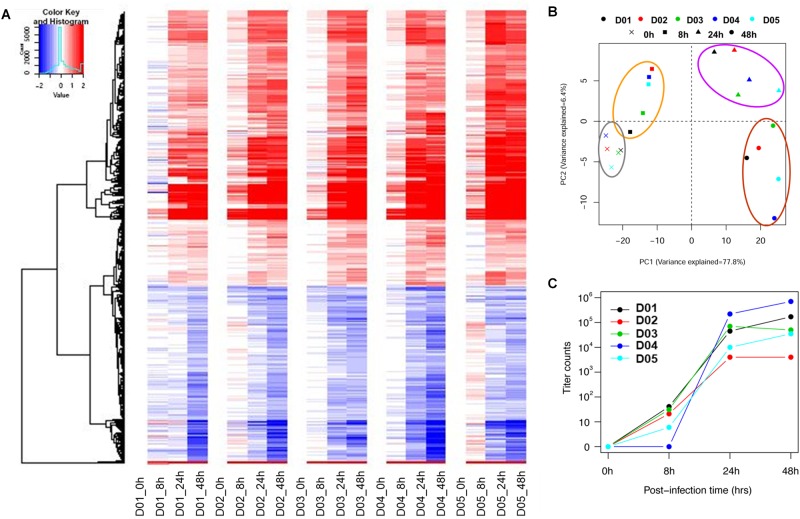
Individual heat-map and PCA analysis of five individual subjects show similar trends but potentially retained some degree of variability in temporal induction and magnitude between donors. **(A)** Individual heat-maps of different subjects revealed largely similar patterns with potential differences in temporal induction from 8 to 48 hpi, including the magnitude of induction. **(B)** Individual PCA plot showed that the transcriptomes across different subjects were not clustered as tightly since the hNEC samples were of different genetic backgrounds. The PCA plot showed a distinct progression of differential gene changes across time-points even at 0 hpi. **(C)** Individual viral titers increased over time to reach high viral loads in samples from all donors. The individual variation was deemed not clinically significant given that a sufficiently high virus titer was attained to induce a consistent response.

To establish a common transcriptomic response signature, the post-infection gene expression profiles (8, 24, and 48 hpi) were compared against the baseline profile from the gene expression microarray data for five donors. Figure 1A and Supplementary Figure S1 show that the response of the nasal epithelium of all five donors followed a similar trend over time. There were potentially also slight differences in the temporal induction and magnitude of response genes expressed across individuals, such as type III IFNs (IFNλ/IFNL), IL36γ, IL-1A and ICAM-1. The details of the individual expression profiles of the genes are provided in Supplementary Table S3. The principal component analysis (PCA) of significantly altered genes (FDR < 0.01) (Figure 1B) showed that the changes in gene expression over the course of infection followed a specific pattern over time, with potentially variable responses across donors. In addition, while the range of virus progeny production in different samples varied by as much as a hundred-fold (Figure 1C), it was deemed not clinically significant in the case of H3N2 infection since all samples led to sufficiently high viral titers post-infection to exert the gene expression changes (Granados et al., 2017).

### Influenza Virus Infection of hNECs Leads to Elevated Expression of Both Innate and Adaptive Response Genes, and Reduced Expression of Metabolism Genes

**FIGURE 2:**
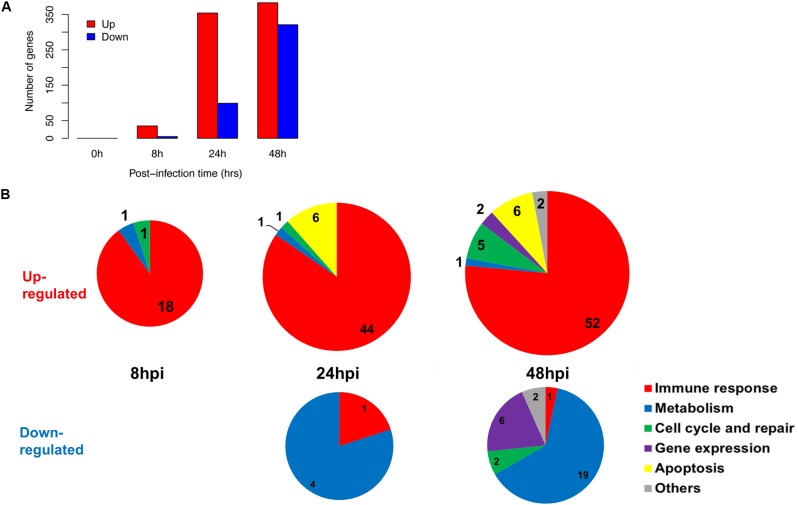
Summary of gene changes in hNECs which correlate with increasing viral titer. **(A)** Gene changes corresponding to increasing viral titer exhibited maximal up-regulation and maximal down-regulation at 48 hpi. **(B)** Temporal analysis demonstrated key up-regulated (upper three pie charts) and down-regulated (bottom two pie charts) gene ontology (GO) terms in the nasal epithelium corresponding to increasing viral titer. The majority of up-regulated GO terms were immune-related, whereas down-regulated GO terms were metabolism-related.

We established that the nasal epithelium interaction with influenza virus was complex but observable *in vitro*, and may reflect the differences in subsequent host response and disease progression. To follow up, we further identified the pathways in which the differentially expressed genes were involved, since their differing expression between individuals may affect these pathways and account for varying susceptibility toward influenza infection. We analyzed the numbers and categories of differentially regulated genes during the increase of viral titers as these genes may constitute the susceptibility factors contributing to differences in pathogenesis. There was an earlier up-regulation of genes at 24 hpi, numbering about 250–350 genes; while the down-regulation of genes was only observed later at 48 hpi (Figure 2A). These genes were subjected to hypergeometric analysis for gene set enrichment based on gene ontology database (*p* < 0.01), and mainly belong to immune response pathways which were up-regulated; and metabolic pathways which were down-regulated following infection (Figure 2B). The analysis revealed that the major pathway changes in the nasal epithelium following influenza infection were predominantly increased antiviral immune responses, and decreased metabolic functions.

### Ingenuity Pathway Analysis Reveals Pathways Initiated at the Nasal Epithelium Following Influenza Infection

**FIGURE 3:**
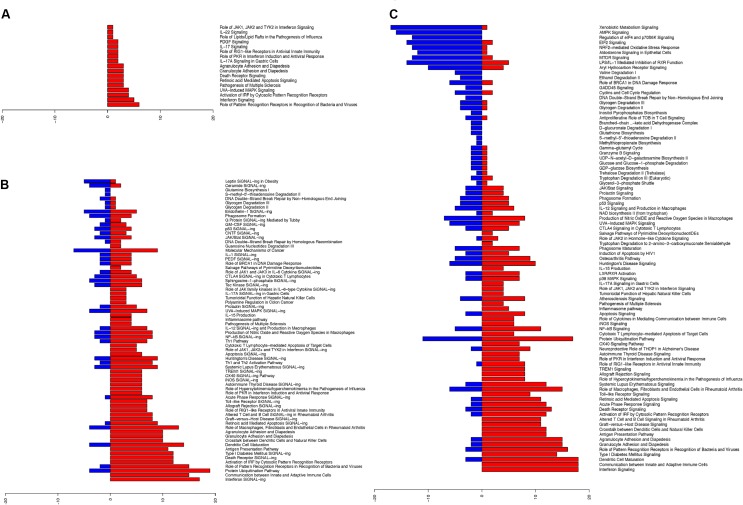
Ingenuity pathway analysis revealed upregulated changes of pathways in hNECs over time and became more diverse. The significant pathway changes following influenza infection and the number of genes (x-axis) significantly up-regulated (red) or down-regulated (blue) in each pathway are shown. **(A)** At 8 hpi, all significant pathways (*p* < 0.05) exhibited up-regulation of components which are all immune-related. **(B)** At 24 hpi, a combination of pathways were significantly altered (*p* < 0.05), with down-regulation of certain biosynthesis pathways. **(C)** At 48 hpi, more of the significant pathways (*p* < 0.05) were down-regulated, while immune response genes shifted toward cytokine production and cross-talk with adaptive immune cells.

Upon establishing the nasal changes following influenza infection, we further categorized these pathways using Ingenuity pathway analysis (IPA) software (Figures 3A–C; Supplementary Tables S4A–C). From the analysis of canonical pathways using genes at FDR < 0.01, the major up-regulated pathways (*p* < 0.05) involved in the nasal epithelium gradually shifted from antiviral responses and inflammasome activation (Figure 3A), to early adaptive immune responses involving antigen presentation and suppression of homeostatic activities (Figures 3B,C). On the other hand, downregulation was observed in DNA damage pathways, as well as in metabolic and biosynthetic pathways (*p* < 0.05) (Figure 3C).

### Comparison of hNEC Infection With Other *in vitro* and Human Studies Indicate That hNEC Responses Closely Mimic *in vivo* Systems

**FIGURE 4:**
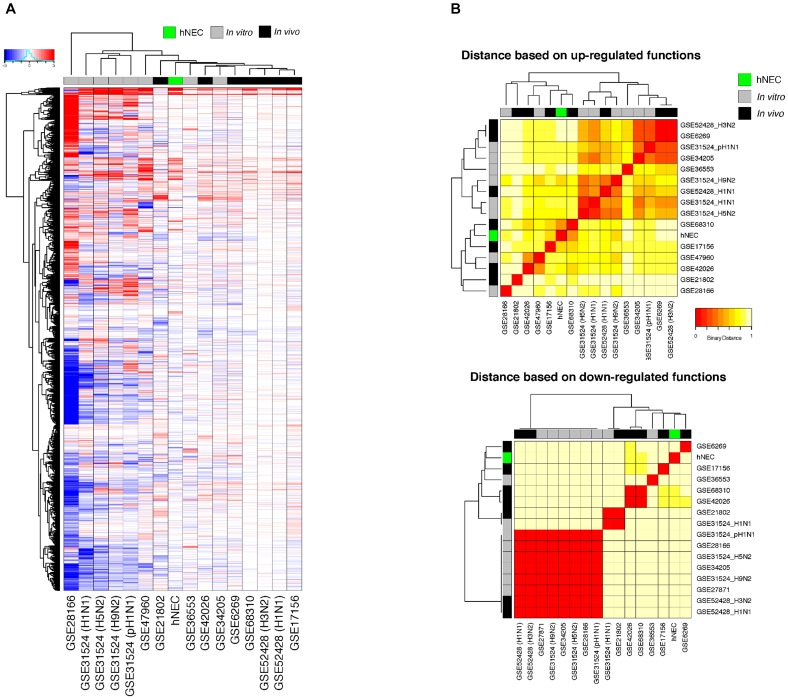
Meta-analysis of genes and functions across *in vitro* and *in vivo* influenza transcriptomic studies at peak responses reveal similarities of hNECs to human studies. **(A)** Transcriptomic signatures (FDR < 0.01) revealed that the differential changes in infected hNECs were more similar to human studies due to heterogeneity of hNECs. **(B)** Using functional clustering of meta-genomic data, hNECs were observed to tightly cluster functionally with most human studies (*in vivo*) at the peak of viral-induced changes of each individual study.

We compared the transcriptomic responses of our infected hNECs with 15 other *in vitro* and *in vivo* influenza infection transcriptomic studies, at their peak responses against influenza. Across all studies, the transcriptome data at the peak of infection were compared against the corresponding baseline data, and the transcriptomic changes were compared across the studies, with FDR < 0.01 being assigned as the baseline for significance (see section “Materials and Methods”). The differential transcriptome signature in hNECs was highly similar to the signatures from other influenza infection models (Figure 4A). Interestingly, compared to the homogenous cell lines tested during *in vitro* studies, our heterogenous hNEC model exhibited a more comparable response to the clinical influenza studies. This is shown by hierarchical clustering where the hNECs are clustered closer to clinical studies in their responses against influenza infection. Furthermore, when the studies were clustered according to functional changes arising from the transcriptomic changes, our hNEC model was also found to cluster closer to large-scale clinical influenza studies based on blood or peripheral blood mononuclear cells (PBMCs), rather than with cell line studies (Figure 4B and Supplementary Figure S2).

### Functional Comparison of Infected hNEC Responses With Other Infection Models Reveals Key Factors Initiated by the Nasal Epithelium

We further compared the hypergeometric gene ontology (GO) analysis of hNECs with those in other studies to identify functional changes associated with the transcriptomic changes elicited by the nasal epithelium, using a cutoff of *p* < 0.01 to elucidate the common and unique pathways with greater confidence (Supplementary Table S5). Our model representing the human nasal epithelium displayed transcriptomic changes that mainly clustered with immune responses and metabolic processes. Compared to the other studies, the infected hNEC model induced comparable increases (FDR_up < 0.01) in immune responses (highlighted in red in Supplementary Table S5), i.e., 47 out of 67 significant immune functions that overlapped with at least one other study, and 37 out of 41 with at least 3 other studies. Therefore, this strong overlap highlights that most immune processes against influenza are locally initiated at the nasal epithelium, which are subsequently propagated, and can be detected systemically. Furthermore, given that they are the primary target cells for influenza infection, the hNECs also exhibited reduction (FDR_down < 0.01) in metabolic processes (highlighted in blue in Supplementary Table S5). The overlap in this reduction was 20 out of 32 with at least one other study; and 6 out of 8 with at least 3 other studies. These reductions in nitrogen compounds, carbohydrates and lipids may be a direct consequence of the infection; or may be part of a signaling mechanism in antiviral responses, which remain to be further explored. On the other hand, the infected hNEC model does not reflect most changes at the cell cycle and tissue repair pathways (highlighted in green in Supplementary Table S5; 11 out of 73), which are likely to be initiated only upon viral clearance. There was also only slight overlap in gene expression mechanisms (highlighted in purple in Supplementary Table S5), i.e., 3 out of 37.

### Quantitative Real-Time PCR Verification With Independent hNEC Infections to Identify Key and Novel Pathways in Influenza Infection

**FIGURE 5:**
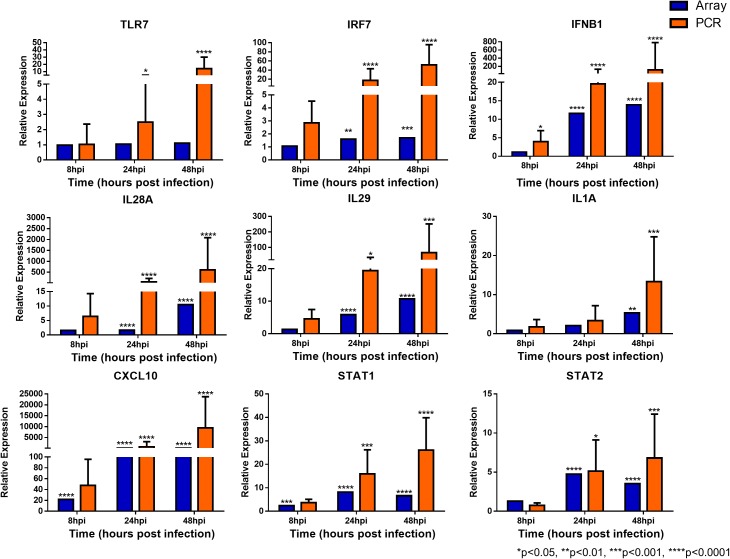
Expression of immune response genes in nasal epithelial cells following influenza infection. Expression of host genes in response to influenza in sequence: TLR7, IRF7, IFNB1, IL28A, IL29, IL1A, CXCL10, STAT1, and STAT2 (*n* = 4–12). The statistical significance level of the RT-qPCR expression was calculated using one-way ANOVA, nonparametric, grouped, Dunn multiple comparison test, with the *p*-value derived by comparison against uninfected control cells. ^∗^*p* < 0.05; ^∗∗^*p* < 0.01; ^∗∗∗^*p* < 0.001; ^∗∗∗∗^*p* < 0.0001. The RT-qPCR data were presented as medians with inter-quartile range.

**FIGURE 6:**
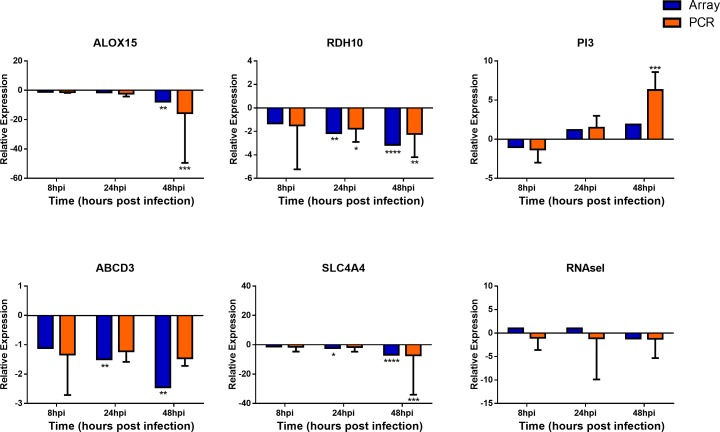
Expression of homeostatic and metabolic genes in nasal epithelial cells following influenza infection. Expression of host genes in response to influenza in sequence: ALOX15, RDH10, PI3, ABCD3, SLC4A4, and RNASel (*n* = 4–12). The statistical significance level of the RT-qPCR expression was calculated using one-way ANOVA, nonparametric, grouped, Dunn multiple comparison test, with the *p*-value derived by comparison against uninfected control cells. ^∗^*p* < 0.05; ^∗∗^*p* < 0.01; ^∗∗∗^*p* < 0.001; ^∗∗∗∗^*p* < 0.0001. The RT-qPCR data were presented as medians and inter-quartile range.

To confirm the validity of the microarray analysis and the similarity of hNEC responses to *in vivo* clinical models, we performed independent infection experiments on another batch of hNEC donors to compare their responses to the transcriptomic analysis. By selecting genes involved in viral sensing, interferon (IFN), antiviral, type-1 inflammatory response, metabolic and homeostatic pathways, we compared the expression profiles between the microarray and the independent infections. In the independent infections, the qPCR analyses revealed close agreement with the microarray expression, and confirmed the expression of viral sensing via TLR7, which activates the JAK-STAT pathways via type I and type III IFN activation to initiate type-1 inflammatory responses (Figure 5). Additionally, antiviral genes were also activated during the infection process with the exception of MUC5AC (Supplementary Figure S3), which concurs with our previous finding where influenza does not alter MUC5AC expression (Yan et al., 2016). Furthermore, we also analyzed genes involved in homeostatic and metabolic activities (Figure 6), and observed reduced expression of genes governing lipid metabolism (ALOX15), retinoic acid synthesis (RDH10), peroxisome transport (ABCD3), and pH regulation (SLC4A4). Interestingly, the mRNA encoding peptidase inhibitor 3 (PI3), which plays a role in anti-bacterial and anti-fungal activity, was up-regulated in the nasal epithelium, implying that this gene may help to prevent secondary bacterial infection.

## Discussion

Evidence is accumulating that the nasal epithelium, the first contact site of respiratory viruses, plays crucial defense functions beyond being host cells for influenza infection. Many studies indicate that the nasal epithelium actively triggers innate immune responses and also modulates adaptive immunity against these viruses (Vareille et al., 2011; Braciale et al., 2012; Yan et al., 2016). However, the scarcity of human nasal cell models impeded in-depth studies on this aspect. The role of nasal epithelium was also unclear since most human studies focused on systemic signals from the blood (Bermejo-Martin et al., 2010; Herberg et al., 2013; Zhai et al., 2015); thereby masking the immune effects of the nasal epithelium and their utility in clinical settings. Therefore, using our hNEC culture differentiated from enriched nasal stem cells, we elucidated the initial changes in the infected nasal epithelium that likely generate most of the signals that ultimately lead to the cascading immune responses mediated by immune cells. Since the nasal epithelium can mount distinct responses that eventually lead to type-1 and type-2 inflammatory responses against influenza, such responses may be crucial in controlling viral pathogenesis and in modulating local antiviral responses (Tan et al., 2017; Edwards et al., 2018).

Our study evaluated nasal epithelial cells differentiated from five different donors, which mounted a consistent response against influenza, with potential differential responses between individuals in antiviral response genes such as IFNλs, IL36γ, IL-1A, and ICAM-1. These genes, while not strongly induced, may play roles in modulating the immune defense against influenza infection to prevent damage to the surrounding tissues (Galani et al., 2017; Klinkhammer et al., 2018; Wein et al., 2018). We also observed that, in the absence of immune cells, the nasal epithelial response skewed heavily toward type-1 inflammatory activation with minimal type-2 inflammatory activating responses. This suggests that naïve epithelial cells primarily evoke a type-1 inflammatory response against invading viruses, especially in the context of influenza. Furthermore, the nasal cells could initiate cross-talk between innate and adaptive immunity via robust production of adaptive immune-activating cytokines and chemokines such as IL-6, CXCL10, and IFNλs including IFNL2 (IL28A) and IFNL1 (IL29). Compared with the other clinical studies involving peripheral blood mononuclear cells (PBMCs), blood or serum from patients, the hNECs exhibited similar transcriptomic changes in up-regulated immune responses, thus identifying nasal-initiated immune functions (JAK-STAT-mediated type-1 inflammatory response activation).

On the other hand, there were critical down-regulated functions in hNECs related to multiple metabolic and DNA damage responses against influenza that were mostly not observed in blood or serum samples (Ivan et al., 2012, 2013; Raval and Nikolajczyk, 2013; Li et al., 2015; Cui et al., 2016). Such reductions in metabolic functions and related metabolites at the primary infection site of influenza may be an interesting area for future investigation to understand their relationships with viral replication and immune functions (Cui et al., 2016; Gonzalez Plaza et al., 2016; Boergeling and Ludwig, 2017; Cui et al., 2017; Smallwood et al., 2017). In addition, these changes in the metabolic and homeostatic pathways are unique to the nasal epithelium, and were not documented in other *in vitro* and clinical studies. Dampening of these homeostatic pathways may impact downstream immune and non-immune responses or cause complications that contribute to the pathogenesis of influenza or other metabolic disorders (Gonzalez Plaza et al., 2016; Smallwood et al., 2017). One such example of altered gene expression related to lipid and peroxisomal metabolism during influenza (Tanner et al., 2014), was also revealed in our study, i.e., ABCD3. Moreover, certain homeostatic proteins serve dual roles, such as BPIFA1 and CD151, which possess pro- or anti-viral, homeostatic and signaling functions in the airway (Akram et al., 2018; Kim et al., 2018; Qiao et al., 2018). Therefore, this study highlighted perturbations in homeostatic and metabolic pathways which warrant further exploration into their underlying mechanisms associated with influenza pathogenesis.

The comparable responses of hNECs with clinical datasets suggest that most influenza-related responses can be detected within the nasal eptithelium. Furthermore, the transcriptomic changes of infected nasal epithelial cells revealed differential regulation of all 11 targets (CD38, HERC5, HERC6, IFI6, IFIH1, LGALS3BP, LY6E, MX1, PARP12, RTP4, and ZBP1) previously thought to be influenza-specific signatures (Andres-Terre et al., 2015). Hence, the fact that these key transcriptomic signatures during influenza were nasal-initiated, underscores the clinical utility of the hNEC model. The signature changes revealed in this study can be exploited to identify and verify potential markers that contribute to severe infection via the human nasal transcriptome *in vitro* and *in vivo*. These markers can then be further applied using molecular diagnostics to detect these signatures during early infection to distinguish patients who may progress to severe disease, especially during outbreaks such as the 2009 H1N1 pandemic in which the severity of infection varied widely among patients. For example, markers such as IFNλs represent factors exerting antiviral responses that are less damaging to the surrounding tissues (Davidson et al., 2016; Galani et al., 2017). This is clinically significant in view of the proximity of the nasal epithelium to the infection; hence nasal diagnosis would facilitate more rapid detection of the severity markers compared to blood or serum specimens. In addition, intervention studies can also be performed using this model in studying local antiviral or immunomodulatory compounds. However, this model is not without its limitations. One disadvantage of this *in vitro* model is its inability to achieve complete viral clearance. It is thus unable to study the responses and repair of the nasal epithelium after viral clearance, for which *in vivo* models would still be necessary. Another limitation is that this study only evaluated infection with a H3N2 strain. Given that hNECs infected with currently circulating strains belonging to H1N1 and B subtypes may respond differently, future studies are warranted to establish a more complete picture of influenza infection signatures in the nasal epithelium.

## Conclusion

In conclusion, the nasal epithelium is an active component of initial host responses against influenza infection, and the nasal epithelial-specific transcriptomic changes may significantly influence the downstream immune responses and homeostasis that define the pathology of influenza in different individuals. Epithelial cells from different donors sustained infection differently; thus potential variations in nasal epithelial transcriptomic changes during influenza may result in differential pathogenesis between individuals. While infected nasal epithelial cells clearly elicited immune responses, the microarray analyses revealed that metabolism and antioxidant processes were also enriched among the transcriptomic changes. Future studies are warranted in the population to investigate nasal-associated factors identified in this study to ensure reproducibility of these factors in contributing to differential influenza pathogenesis amongst different individuals. This can be achieved through replicate infection studies of cells obtained from the same individual. In addition, being the site of closest proximity to the infection as well as exerting most responses observed in blood/serum may encourage the development of rapid molecular diagnostic tools based on the detection of nasal epithelial signatures that may predict the severity of influenza.

## Author Contributions

KT, YY, HC, TT, RS, VC, and DW conceived and designed the study, including selection of online transcriptomic datasets. KT, YY, WK, LL, and HC carried out the experiments and bioinformatics analysis and performed the statistical analyses. KT, HC, and VC co-wrote the original draft of the paper. All authors contributed to data interpretation, reviewed and edited the drafts, and approved the final version for submission.

## Conflict of Interest Statement

The authors declare that the research was conducted in the absence of any commercial or financial relationships that could be construed as a potential conflict of interest.

## Supplementary Material

FIGURE S1 Individual heat-maps of all differentially expressed genes following influenza infection of five donor hNECs.

FIGURE S2 Meta-analysis and hierarchical functions across *in vitro* and *in vivo* influenza transcriptomic studies.

FIGURE S3 Expression of host response genes in nasal epithelium following influenza infection.

TABLE S1 Information and source of hNECs from five donors.

TABLE S2 Details of transcriptomic studies included in meta-analysis.

TABLE S3 Complete gene list output from microarray analysis.

TABLE S4 Canonical pathways of the nasal epithelium following influenza virus infection. **(A)** 8 hpi; **(B)** 24 hpi; **(C)** 48 hpi.

TABLE S5 Functional pathways (gene ontology terms) that are differentially regulated across all 15 studies analyzed.
