# Supplementary figures and images for: Comparative Transcriptomic and Metagenomic Analyses of Influenza Virus-Infected Nasal Epithelial Cells From Multiple Individuals Reveal Specific Nasal-Initiated Signatures
Source: Front Microbiol. 2018 Nov 14;9:2685. doi: 10.3389/fmicb.2018.02685 (PMC6246735; doi:10.3389/fmicb.2018.02685)

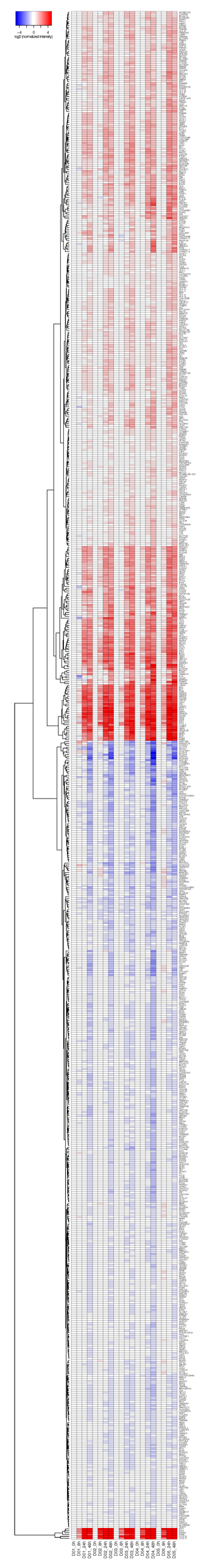

Supplement: FIGURE S1 — Individual heat-maps of all differentially expressed genes following influenza infection of five donor hNECs. [file Image_1.TIF]

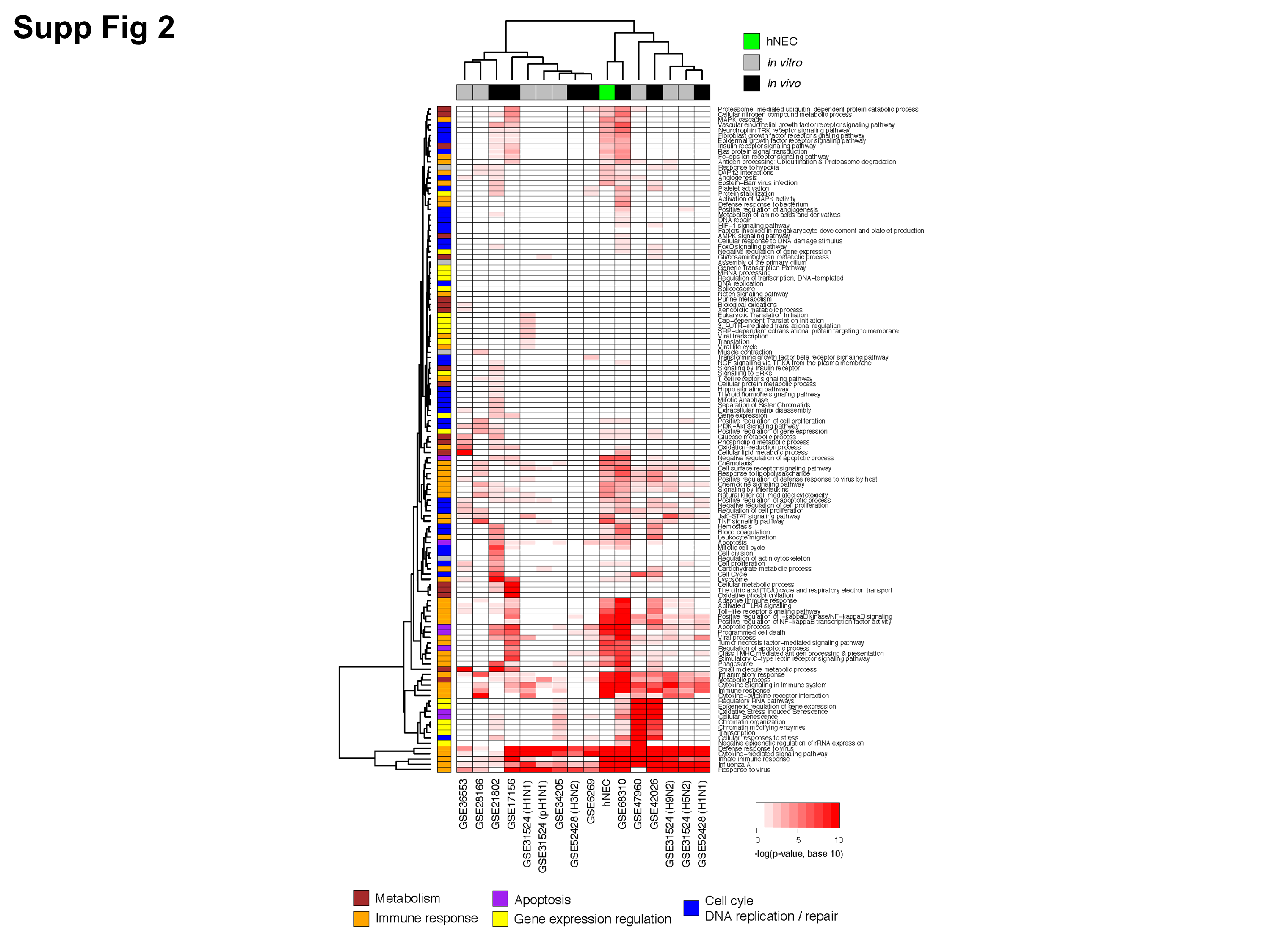

Supplement: FIGURE S2 — Meta-analysis and hierarchical functions across in vitro and in vivo influenza transcriptomic studies. [file Image_2.TIF]

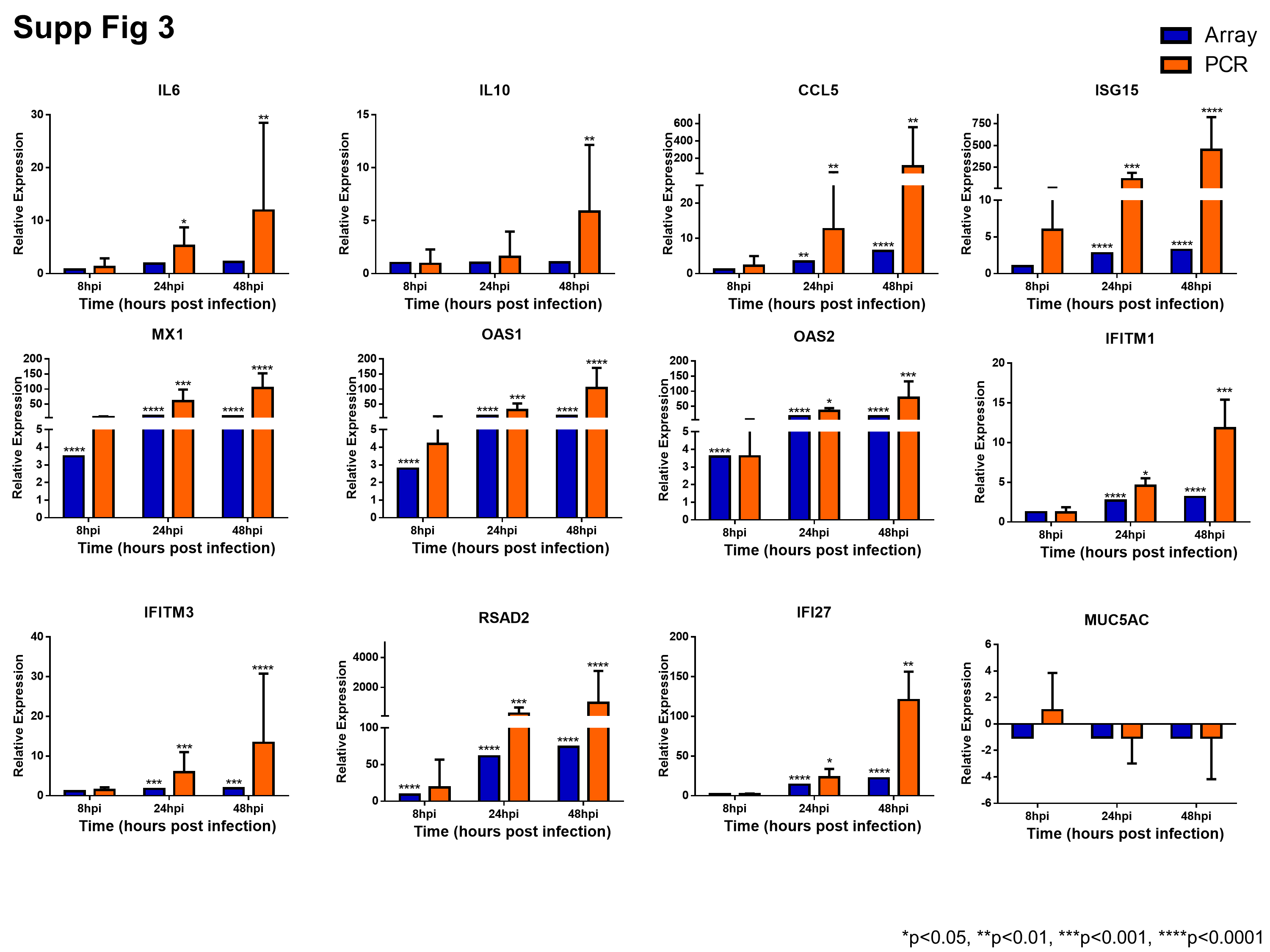

Supplement: FIGURE S3 — Expression of host response genes in nasal epithelium following influenza infection. [file Image_3.TIF]
