# Supplementary material for: Comparative Transcriptomic and Metagenomic Analyses of Influenza Virus-Infected Nasal Epithelial Cells From Multiple Individuals Reveal Specific Nasal-Initiated Signatures
Source: Front Microbiol. 2018 Nov 14;9:2685. doi: 10.3389/fmicb.2018.02685 (PMC6246735; doi:10.3389/fmicb.2018.02685)
Supplement: TABLE S1 — Information and source of hNECs from five donors. [file Table_1.DOCX]

**Table S1. Donor information of 5 hNECs sources**

| **Sample ID** | **Age** | **Gender** | **Diagnosis** | **Sample** | **Smoking history** | **Drug treatment*** |
| --- | --- | --- | --- | --- | --- | --- |
| D01 | 22 | M | SD, AR | IT | Non-smoker | no |
| D02 | 32 | M | SD | IT | Non-smoker | no |
| D03 | 35 | M | SD | IT | Non-smoker | no |
| D04 | 51 | M | SD | IT | Non-smoker | no |
| D05 | 23 | F | SD | IT | Non-smoker | no |

Abbreviations: AR, allergic rhinitis; SD, septal deviation; IT, inferior turbinate;

*, corticosteroids and antibiotics within 3 months before the surgery
