# Supplementary material for: Comparative Transcriptomic and Metagenomic Analyses of Influenza Virus-Infected Nasal Epithelial Cells From Multiple Individuals Reveal Specific Nasal-Initiated Signatures
Source: Front Microbiol. 2018 Nov 14;9:2685. doi: 10.3389/fmicb.2018.02685 (PMC6246735; doi:10.3389/fmicb.2018.02685)
Supplement: TABLE S2 — Details of transcriptomic studies included in meta-analysis. [file Table_2.DOCX]

**Table S2. Details of transcriptomic studies used in meta-analysis**

| **GEO accession** | **Study** | **Journal** | **Infection Type** | **Study Design** | **Sample size** | Sample Type | Type |
| --- | --- | --- | --- | --- | --- | --- | --- |
| **GSE6269** | Ramilo et al | Blood 2007 | influenza A | acute infections, pediatric | 18 flu, 6 control | PBMC | Blood |
| **GSE17156** | Zaas et al | Cell Host & Microbe 2009 | influenza A |  | 8 symptomatic | PBMC | Blood |
| **GSE21802** | Bermejo-Martin et al | Critical Care | H1N1 |  | 19 patients, with multiple runs from different dates | serum | Blood |
| **GSE42026** | Herberg et al | JID | H1N1/09 |  | 19 flu, 33 controls | whole blood | Blood |
| **GSE52428** | Woods et al | PLoS One | H1N1 |  | 12 symptomatic | PBMC | Blood |
| **GSE52428** | Woods et al | PLoS One | H3N2 |  | 9 symptomatic | PBMC | Blood |
| **GSE68310** | Zhai et al |  | Influenza A | Prospective cohort | 37 flu A patients | PBMC | Blood |
| **GSE31524** | Sutejo et al | PLoS One | H1N1 (10H) |  |  | A549 | In vitro |
| **GSE31524** | Sutejo et al | PLoS One | H5N2-F118 |  |  | A549 | In vitro |
| **GSE31524** | Sutejo et al | PLoS One | H9N2 |  |  | A549 | In vitro |
| **GSE31524** | Sutejo et al | PLoS One | pandemic H1N1 |  |  | A549 | In vitro |
| **GSE34205** | Ioannidis et al | JVI | H1N1 A/WSN/33; H3N2 A/Udorn/72 |  | 28 flu, 12 control | hAEC culture from non-cystic fibrosis patients | In vitro |
| **GSE36553** | Loveday et al | JVI | H1N1A/Mexico/InDRE4487/2009;H7N7/A/chicken/Germany/R28/2003 |  |  | A549 | In vitro |
| **GSE47960** | Mitchell et al |  | Influenza A (H5N1 VN1203; H1N1 CA04; H1N1 NL602) |  |  | Calu3 | In vitro |
| **GSE28166** | Li et al | JVI | H5N1 Avian flu | HBE |  | Calu3 | In vitro |
