# Supplementary material for: Comparative Transcriptomic and Metagenomic Analyses of Influenza Virus-Infected Nasal Epithelial Cells From Multiple Individuals Reveal Specific Nasal-Initiated Signatures
Source: Front Microbiol. 2018 Nov 14;9:2685. doi: 10.3389/fmicb.2018.02685 (PMC6246735; doi:10.3389/fmicb.2018.02685)
Supplement: TABLE S4 — Canonical pathways of the nasal epithelium following influenza virus infection. (A) 8 hpi; (B) 24 hpi; (C) 48 hpi. [file Table_4.docx]

**Table S4. Canonical pathways of the nasal epithelium post influenza infection**

**A. 8hpi**

| Ingenuity Canonical Pathways | -log(p-value) | Ratio | z-score | Molecules | |  |  |
| --- | --- | --- | --- | --- | --- | --- | --- |
| Interferon Signaling | 7.94E+00 | 1.39E-01 | 2.236 | IFIT3↑,IFIT1↑,OAS1↑,MX1↑,STAT1↑ | | | |
| Role of Pattern Recognition Receptors in Recognition of Bacteria and Viruses | 6.51E+00 | 4.58E-02 | NaN | IFIH1↑,OAS1↑,OAS2↑,DDX58↑,EIF2AK2↑,OAS3↑ | | | |
| Pathogenesis of Multiple Sclerosis | 6.14E+00 | 3.33E-01 | NaN | CXCL10↑,CXCL11↑,CXCL9↑ | | |  |
| Activation of IRF by Cytosolic Pattern Recognition Receptors | 5.09E+00 | 6.56E-02 | 2 | IFIH1↑,DDX58↑,IFIT2↑,STAT1↑ | | | |
| UVA-Induced MAPK Signaling | 4.16E+00 | 3.81E-02 | 2 | PARP12↑,STAT1↑,PARP9↑,PARP14↑ | | | |
| Retinoic acid Mediated Apoptosis Signaling | 3.59E+00 | 5.08E-02 | NaN | PARP12↑,PARP9↑,PARP14↑ | | |  |
| Death Receptor Signaling | 3.02E+00 | 3.26E-02 | NaN | PARP12↑,PARP9↑,PARP14↑ | | |  |
| IL-17A Signaling in Gastric Cells | 2.90E+00 | 8.00E-02 | NaN | CXCL10↑,CXCL11↑ | |  |  |
| Role of PKR in Interferon Induction and Antiviral Response | 2.47E+00 | 4.88E-02 | NaN | EIF2AK2↑,STAT1↑ | |  |  |
| Role of RIG1-like Receptors in Antiviral Innate Immunity | 2.45E+00 | 4.76E-02 | NaN | IFIH1↑,DDX58↑ | |  |  |
| Granulocyte Adhesion and Diapedesis | 2.29E+00 | 1.81E-02 | NaN | CXCL10↑,CXCL11↑,CXCL9↑ | | |  |
| Agranulocyte Adhesion and Diapedesis | 2.22E+00 | 1.70E-02 | NaN | CXCL10↑,CXCL11↑,CXCL9↑ | | |  |
| IL-17 Signaling | 1.86E+00 | 2.35E-02 | NaN | CXCL10↑,CXCL11↑ | |  |  |
| PDGF Signaling | 1.81E+00 | 2.22E-02 | NaN | EIF2AK2↑,STAT1↑ | |  |  |
| Role of Lipids/Lipid Rafts in the Pathogenesis of Influenza | 1.38E+00 | 5.00E-02 | NaN | RSAD2↑ |  |  |  |
| IL-22 Signaling | 1.31E+00 | 4.17E-02 | NaN | STAT1↑ |  |  |  |
| Role of JAK1, JAK2 and TYK2 in Interferon Signaling | 1.31E+00 | 4.17E-02 | NaN | STAT1↑ |  |  |  |

**B. 24 hpi**

| Ingenuity Canonical Pathways | -log(p-value) | Ratio | z-score | Molecules |
| --- | --- | --- | --- | --- |
| Interferon SiGNAL↓ing | 1.71E+01 | 4.72E-01 | 3.638 | SOCS1↑,IFIT3↑,OAS1↑,MX1↑,IFNB1↑,IFI35↑,PSMB8↑,JAK2↑,TAP1↑,IRF1↑,ISG15↑,IFITM3↑,IFIT1↑,STAT2↑,IFI6↑,STAT1↑,IFITM1↑ |
| Antigen Presentation Pathway | 8.56E+00 | 2.89E-01 | NaN | B2M↑,HLA-G↑,PSMB9↑,NLRC5↑,HLA-C↑,HLA-B↑,PSMB8↑,HLA-F↑,TAP1↑,TAP2↑,HLA-E↑ |
| Communication between Innate and Adaptive Immune Cells | 8.04E+00 | 1.72E-01 | NaN | B2M↑,HLA-B↑,IFNB1↑,CCL5↑,TLR2↑,CXCL10↑,HLA-G↑,IL36G↑,IL1RN↑,HLA-C↑,TNFSF13↑,TLR3↑,HLA-F↑,TNFSF13B↑,HLA-E↑ |
| Activation of IRF by Cytosolic Pattern Recognition Receptors | 7.21E+00 | 1.97E-01 | 1.732 | IFIH1↑,DHX58↑,IRF7↑,ZBP1↑,DDX58↑,IFNB1↑,IKBKE↑,STAT2↑,IFIT2↑,ADAR↑,STAT1↑,ISG15↑ |
| Role of Pattern Recognition Receptors in Recognition of Bacteria and Viruses | 7.11E+00 | 1.30E-01 | 2.496 | OAS1↑,OAS2↑,MYD88↑,IFNB1↑,CCL5↑,OAS3↑,TLR2↑,IFIH1↑,CLEC7A↑,IRF7↑,DDX58↑,CASP1↑,PIK3CB↓,EIF2AK2↑,TLR3↑,RIPK2↑,ATM↓ |
| Protein Ubiquitination Pathway | 6.14E+00 | 8.71E-02 | NaN | B2M↑,PSMB9↑,USP18↑,USP15↑,UBE4B↓,USP41↑,PSMB10↑,HLA-B↑,PSME2↑,PSMB8↑,DNAJA1↑,UBE2L6↑,TAP1↑,USP3↑,HLA-C↑,UBE2G1↓,PSMA4↑,USP46↓,NEDD4L↓,PSMA2↑,TAP2↑,BIRC3↑,USP25↑ |
| Dendritic Cell Maturation | 5.64E+00 | 9.78E-02 | 2.183 | B2M↑,FCGR2C↑,MYD88↑,HLA-B↑,IFNB1↑,IKBKE↑,JAK2↑,TLR2↑,IL36G↑,PLCB4↓,PLCE1↓,HLA-C↑,IL1RN↑,STAT2↑,PIK3CB↓,TLR3↑,STAT1↑,ATM↓ |
| Retinoic acid Mediated Apoptosis SiGNAL↓ing | 5.49E+00 | 1.69E-01 | 2.53 | PARP10↑,IFNB1↑,ZC3HAV1↑,PARP8↑,TNFSF10↑,TNFRSF10D↓,PARP12↑,PARP9↑,IRF1↑,PARP14↑ |
| Death Receptor SiGNAL↓ing | 5.22E+00 | 1.30E-01 | 2.887 | PARP10↑,ZC3HAV1↑,PARP8↑,TNFSF10↑,IKBKE↑,PARP12↑,BIRC3↑,PARP9↑,CASP7↑,FAS↑,PARP14↑,CASP10↑ |
| Graft-versus-Host Disease SiGNAL↓ing | 4.73E+00 | 1.82E-01 | NaN | HLA-G↑,IL36G↑,HLA-C↑,IL1RN↑,HLA-B↑,HLA-F↑,FAS↑,HLA-E↑ |
| Type I Diabetes Mellitus SiGNAL↓ing | 4.54E+00 | 1.12E-01 | 1.89 | HLA-G↑,SOCS1↑,HLA-C↑,MYD88↑,HLA-B↑,IKBKE↑,JAK2↑,HLA-F↑,STAT1↑,FAS↑,HLA-E↑,IRF1↑ |
| Pathogenesis of Multiple Sclerosis | 4.25E+00 | 4.44E-01 | NaN | CXCL10↑,CXCL11↑,CCL5↑,CXCL9↑ |
| Role of RIG1-like Receptors in Antiviral Innate Immunity | 3.95E+00 | 1.67E-01 | 1.89 | IFIH1↑,DHX58↑,IRF7↑,DDX58↑,IFNB1↑,IKBKE↑,CASP10↑ |
| UVA-Induced MAPK SiGNAL↓ing | 3.94E+00 | 1.05E-01 | 0.905 | PLCB4↓,PLCE1↓,PARP10↑,ZC3HAV1↑,PARP8↑,PIK3CB↓,PARP12↑,STAT1↑,PARP9↑,PARP14↑,ATM↓ |
| Crosstalk between Dendritic Cells and Natural Killer Cells | 3.89E+00 | 1.12E-01 | NaN | HLA-G↑,IL15RA↑,HLA-C↑,HLA-B↑,IFNB1↑,TNFSF10↑,TLR3↑,HLA-F↑,FAS↑,HLA-E↑ |
| Allograft Rejection SiGNAL↓ing | 3.58E+00 | 1.46E-01 | NaN | B2M↑,HLA-G↑,HLA-C↑,HLA-B↑,HLA-F↑,FAS↑,HLA-E↑ |
| Role of JAK1, JAK2↑ and TYK2 in Interferon SiGNAL↓ing | 3.43E+00 | 2.08E-01 | NaN | SOCS1↑,IFNB1↑,STAT2↑,JAK2↑,STAT1↑ |
| Role of PKR in Interferon Induction and Antiviral Response | 3.15E+00 | 1.46E-01 | NaN | IFNB1↑,IKBKE↑,TLR3↑,EIF2AK2↑,STAT1↑,IRF1↑ |
| Role of Hypercytokinemia/hyperchemokinemia in the Pathogenesis of Influenza | 3.15E+00 | 1.46E-01 | NaN | CXCL10↑,IL36G↑,IL1RN↑,IFNB1↑,CCL5↑,IFNL1↑ |
| Autoimmune Thyroid Disease SiGNAL↓ing | 3.09E+00 | 1.43E-01 | NaN | HLA-G↑,HLA-C↑,HLA-B↑,HLA-F↑,FAS↑,HLA-E↑ |
| iNOS SiGNAL↓ing | 2.98E+00 | 1.36E-01 | 2.236 | MYD88↑,IKBKE↑,JAK2↑,STAT1↑,IRF1↑,IRAK2↑ |
| Cytotoxic T Lymphocyte-mediated Apoptosis of Target Cells | 2.90E+00 | 1.61E-01 | NaN | B2M↑,HLA-C↑,HLA-B↑,CASP7↑,FAS↑ |
| Altered T Cell and B Cell SiGNAL↓ing in Rheumatoid Arthritis | 2.78E+00 | 9.64E-02 | NaN | TLR2↑,IL36G↑,IL1RN↑,TNFSF13↑,HLA-B↑,TLR3↑,FAS↑,TNFSF13B↑ |
| Sphingosine-1-phosphate SiGNAL↓ing | 2.74E+00 | 8.06E-02 | -2.333 | ASAH2B↑,S1PR3↑,PLCB4↓,PLCE1↓,CASP1↑,PIK3CB↓,CASP4↑,CASP7↑,ATM↓,CASP10↑ |
| Inflammasome pathway | 2.68E+00 | 1.90E-01 | NaN | AIM2↑,MYD88↑,CASP1↑,NEK7↑ |
| Role of Macrophages, Fibroblasts and Endothelial Cells in Rheumatoid Arthritis | 2.49E+00 | 5.63E-02 | NaN | SOCS1↑,TCF4↑,MYD88↑,MMP13↑,IKBKE↑,CCL5↑,JAK2↑,TLR2↑,IL36G↑,PLCB4↓,PLCE1↓,IL1RN↑,PIK3CB↓,TLR3↑,TNFSF13B↑,IRAK2↑,ATM↓ |
| OX40 SiGNAL↓ing Pathway | 2.43E+00 | 1.07E-01 | NaN | B2M↑,HLA-G↑,HLA-C↑,HLA-B↑,HLA-F↑,HLA-E↑ |
| Toll-like Receptor SiGNAL↓ing | 2.41E+00 | 9.33E-02 | 2.646 | TLR2↑,IL36G↑,MYD88↑,IL1RN↑,TLR3↑,EIF2AK2↑,IRAK2↑ |
| CTLA4 SiGNAL↓ing in Cytotoxic T Lymphocytes | 2.33E+00 | 8.16E-02 | NaN | B2M↑,HLA-C↑,PPM1J↑,HLA-B↑,PPM1L↓,PIK3CB↓,JAK2↑,ATM↓ |
| DNA Double-Strand Break Repair by Homologous Recombination | 2.26E+00 | 2.14E-01 | NaN | BRCA2↑,NBN↑,ATM↓ |
| DNA Double-Strand Break Repair by Non-Homologous End Joining | 2.26E+00 | 2.14E-01 | NaN | PRKDC↓,NBN↑,ATM↓ |
| IL-15 Production | 2.21E+00 | 1.43E-01 | NaN | IFNB1↑,JAK2↑,STAT1↑,IRF1↑ |
| Prolactin SiGNAL↓ing | 2.17E+00 | 8.43E-02 | 0 | SOCS1↑,NMI↑,PIK3CB↓,JAK2↑,STAT1↑,IRF1↑,ATM↓ |
| JAK/Stat SiGNAL↓ing | 2.17E+00 | 8.43E-02 | 0.378 | SOCS1↑,PIAS2↓,PIK3CB↓,STAT2↑,JAK2↑,STAT1↑,ATM↓ |
| Th1 Pathway | 2.09E+00 | 6.92E-02 | -0.378 | SOCS1↑,HLA-B↑,LGALS9↑,CD274↑,PIK3CB↓,JAK2↑,STAT1↑,IRF1↑,ATM↓ |
| NF-ÎºB SiGNAL↓ing | 2.07E+00 | 6.18E-02 | 1.508 | TLR2↑,IL36G↑,MYD88↑,IL1RN↑,BMPR2↑,PIK3CB↓,BMPR1B↓,TLR3↑,EIF2AK2↑,TNFSF13B↑,ATM↓ |
| Role of JAK1 and JAK3 in Î³c Cytokine SiGNAL↓ing | 1.99E+00 | 8.70E-02 | NaN | SOCS1↑,IL15RA↑,PIK3CB↓,JAK2↑,STAT1↑,ATM↓ |
| TREM1 SiGNAL↓ing | 1.96E+00 | 8.57E-02 | 2.449 | TLR2↑,NLRC5↑,MYD88↑,CASP1↑,JAK2↑,TLR3↑ |
| Phagosome Formation | 1.90E+00 | 6.90E-02 | NaN | TLR2↑,FCGR2C↑,CLEC7A↑,PLCB4↓,PLCE1↓,PIK3CB↓,TLR3↑,ATM↓ |
| Granulocyte Adhesion and Diapedesis | 1.86E+00 | 6.02E-02 | NaN | CXCL10↑,IL36G↑,CXCL11↑,IL1RN↑,CCL28↑,MMP13↑,CCL22↑,CCL5↑,CXCL9↑,CX3CL1↑ |
| Systemic Lupus Erythematosus SiGNAL↓ing | 1.85E+00 | 5.53E-02 | NaN | HLA-G↑,FCGR2C↑,IL36G↑,HLA-C↑,IL1RN↑,HLA-B↑,PIK3CB↓,PRPF38B↓,HLA-F↑,HLA-E↑,TNFSF13B↑,ATM↓ |
| Production of Nitric Oxide and Reactive Oxygen Species in Macrophages | 1.83E+00 | 5.70E-02 | 1.508 | APOL1↑,TLR2↑,PPM1J↑,PPM1L↓,IKBKE↑,PIK3CB↓,JAK2↑,STAT1↑,IRF1↑,ATM↓,APOD↑ |
| IL-12 SiGNAL↓ing and Production in Macrophages | 1.82E+00 | 6.25E-02 | NaN | APOL1↑,TLR2↑,MYD88↑,IKBKE↑,PIK3CB↓,STAT1↑,IRF1↑,ATM↓,APOD↑ |
| Salvage Pathways of Pyrimidine Deoxyribonucleotides | 1.75E+00 | 2.50E-01 | NaN | TYMP↑,APOBEC3G↑ |
| Role of BRCA1 in DNA Damage Response | 1.75E+00 | 7.69E-02 | 1 | BRCA2↑,STAT1↑,BRIP1↑,NBN↑,PHF10↓,ATM↓ |
| Polyamine Regulation in Colon Cancer | 1.70E+00 | 1.36E-01 | NaN | TCF4↑,MXD1↑,PSME2↑ |
| Agranulocyte Adhesion and Diapedesis | 1.70E+00 | 5.68E-02 | NaN | CXCL10↑,IL36G↑,CXCL11↑,IL1RN↑,CCL28↑,MMP13↑,CCL22↑,CCL5↑,CXCL9↑,CX3CL1↑ |
| CNTF SiGNAL↓ing | 1.66E+00 | 8.33E-02 | 0.447 | LIFR↑,PIK3CB↓,JAK2↑,STAT1↑,ATM↓ |
| Th1 and Th2 Activation Pathway | 1.64E+00 | 5.56E-02 | NaN | SOCS1↑,HLA-B↑,LGALS9↑,BMPR2↑,CD274↑,PIK3CB↓,JAK2↑,STAT1↑,IRF1↑,ATM↓ |
| Tumoricidal Function of Hepatic Natural Killer Cells | 1.60E+00 | 1.25E-01 | NaN | SERPINB9↑,CASP7↑,FAS↑ |
| Leptin SiGNAL↓ing in Obesity | 1.58E+00 | 7.06E-02 | NaN | PLCB4↓,PLCE1↓,PRKAR2A↓,PIK3CB↓,JAK2↑,ATM↓ |
| S-methyl-5'-thioadenosine Degradation II | 1.57E+00 | 1.00E+00 | NaN | MTAP↓ |
| Glutamine Biosynthesis I | 1.57E+00 | 1.00E+00 | NaN | GLUL↓ |
| Endothelin-1 SiGNAL↓ing | 1.55E+00 | 5.38E-02 | 0 | PLCB4↓,PLCE1↓,CASP1↑,RARRES3↑,PIK3CB↓,CASP4↑,CASP7↑,GNAL↓,ATM↓,CASP10↑ |
| IL-17A SiGNAL↓ing in Gastric Cells | 1.55E+00 | 1.20E-01 | NaN | CXCL10↑,CXCL11↑,CCL5↑ |
| Role of JAK family kinases in IL-6-type Cytokine SiGNAL↓ing | 1.55E+00 | 1.20E-01 | NaN | SOCS1↑,JAK2↑,STAT1↑ |
| Huntington's Disease SiGNAL↓ing | 1.55E+00 | 5.00E-02 | -2.828 | TGM2↑,HDAC9↑,GNB4↑,PLCB4↓,PSME2↑,CASP1↑,PIK3CB↓,CASP4↑,NAPA↑,CASP7↑,ATM↓,CASP10↑ |
| PEDF SiGNAL↓ing | 1.54E+00 | 6.90E-02 | 0.816 | TCF4↑,IKBKE↑,PIK3CB↓,CASP7↑,FAS↑,ATM↓ |
| p53 SiGNAL↓ing | 1.53E+00 | 6.31E-02 | 1.134 | PRKDC↓,HDAC9↑,PMAIP1↑,PIK3CB↓,PML↑,FAS↑,ATM↓ |
| Apoptosis SiGNAL↓ing | 1.50E+00 | 6.74E-02 | 0 | IKBKE↑,BCL2A1↑,BIRC3↑,CASP7↑,FAS↑,CASP10↑ |
| Glycogen Degradation II | 1.48E+00 | 1.82E-01 | NaN | TYMP↑,MTAP↓ |
| IL-1 SiGNAL↓ing | 1.44E+00 | 6.52E-02 | 0.447 | GNB4↑,MYD88↑,PRKAR2A↓,IKBKE↑,GNAL↓,IRAK2↑ |
| Tec Kinase SiGNAL↓ing | 1.44E+00 | 5.36E-02 | 0.816 | GNB4↑,TNFSF10↑,PIK3CB↓,STAT2↑,JAK2↑,STAT1↑,GNAL↓,FAS↑,ATM↓ |
| Acute Phase Response SiGNAL↓ing | 1.42E+00 | 5.33E-02 | 0.707 | SOCS1↑,SERPING1↑,IL36G↑,TCF4↑,MYD88↑,IL1RN↑,IKBKE↑,PIK3CB↓,JAK2↑ |
| Ceramide SiGNAL↓ing | 1.42E+00 | 6.45E-02 | 0.816 | S1PR3↑,PPM1J↑,PPM1L↓,PIK3CB↓,CERK↓,ATM↓ |
| Molecular Mechanisms of Cancer | 1.39E+00 | 4.32E-02 | NaN | PRKDC↓,PMAIP1↑,TCF4↑,PRKAR2A↓,BMPR2↑,JAK2↑,BMPR1B↓,FAS↑,NBN↑,PLCB4↓,PIK3CB↓,BIRC3↑,CASP7↑,GNAL↓,CASP10↑,ATM↓ |
| GM-CSF SiGNAL↓ing | 1.34E+00 | 6.85E-02 | 0.447 | PIK3CB↓,JAK2↑,STAT1↑,BCL2A1↑,ATM↓ |
| Glycogen Degradation III | 1.34E+00 | 1.54E-01 | NaN | TYMP↑,MTAP↓ |
| Guanosine Nucleotides Degradation III | 1.34E+00 | 1.54E-01 | NaN | NT5E↑,XDH↑ |
| G Protein SiGNAL↓ing Mediated by Tubby | 1.31E+00 | 9.68E-02 | NaN | GNB4↑,PLCB4↓,JAK2↑ |

**C. 48 hpi**

| Ingenuity Canonical Pathways | -log(p-value) | Ratio | z-score | Molecules |
| --- | --- | --- | --- | --- |
| Interferon Signaling | 1.56E+01 | 5.00E-01 | 4.243 | IFIT3↑,OAS1↑,MX1↑,IFNB1↑,IFI35↑,PSMB8↑,JAK2↑,IFITM2↑,TAP1↑,IRF1↑,BAK1↑,ISG15↑,IFITM3↑,IFIT1↑,STAT2↑,IFI6↑,STAT1↑,IFITM1↑ |
| Communication between Innate and Adaptive Immune Cells | 8.10E+00 | 2.07E-01 | NaN | B2M↑,IL1A↑,HLA-A↑,HLA-B↑,IFNB1↑,CCL5↑,IL33↑,TLR2↑,CXCL10↑,HLA-G↑,IL36G↑,HLA-C↑,IL1RN↑,TNFSF13↑,TLR3↑,HLA-F↑,TNFSF13B↑,HLA-E↑ |
| Antigen Presentation Pathway | 7.80E+00 | 3.16E-01 | NaN | B2M↑,HLA-G↑,PSMB9↑,NLRC5↑,HLA-C↑,HLA-A↑,HLA-B↑,PSMB8↑,HLA-F↑,TAP1↑,TAP2↑,HLA-E↑ |
| Activation of IRF by Cytosolic Pattern Recognition Receptors | 7.04E+00 | 2.30E-01 | 1.069 | DHX58↑,ZBP1↑,IFNB1↑,IKBKE↑,ADAR↑,ISG15↑,IFIH1↑,IRF7↑,DDX58↑,MAVS↓,STAT2↑,IKBKAP↓,IFIT2↑,STAT1↑ |
| Retinoic acid Mediated Apoptosis Signaling | 6.36E+00 | 2.20E-01 | 2.496 | PARP10↑,IFNB1↑,ZC3HAV1↑,TNFSF10↑,PARP8↑,TNFRSF10D↓,PARP12↑,CASP8↑,TNFRSF10A↑,PARP9↑,IRF1↑,PARP14↑,PARP1↓ |
| Death Receptor Signaling | 6.18E+00 | 1.74E-01 | 2.5 | PARP10↑,ZC3HAV1↑,PARP8↑,TNFSF10↑,IKBKE↑,PARP12↑,PARP9↑,FAS↑,ARHGDIB↓,PARP1↓,CASP8↑,BIRC3↑,TNFRSF10A↑,PARP14↑,CASP10↑,HSPB1↓ |
| Graft-versus-Host Disease Signaling | 6.05E+00 | 2.50E-01 | NaN | IL33↑,HLA-G↑,IL36G↑,IL1A↑,HLA-C↑,HLA-A↑,IL1RN↑,HLA-B↑,HLA-F↑,FAS↑,HLA-E↑ |
| Role of Pattern Recognition Receptors in Recognition of Bacteria and Viruses | 5.95E+00 | 1.45E-01 | 2.138 | IL1A↑,OAS1↑,OAS2↑,MYD88↑,IFNB1↑,CCL5↑,OAS3↑,TLR2↑,IFIH1↑,CLEC7A↑,IRF7↑,DDX58↑,CASP1↑,MAVS↓,EIF2AK2↑,TLR3↑,RIPK2↑,ATM↓,PRKCA↓ |
| Protein Ubiquitination Pathway | 5.57E+00 | 1.06E-01 | NaN | B2M↑,USP18↑,USP41↑,HLA-A↑,HLA-B↑, DNAJC10↓, PSMB8↑, DNAJA1↑, TAP1↑, USP3↓,DNAJC4↓,NEDD4L↓,PSMA2↑,BIRC3↑,AMFR↓,HSPA4L↓,PSMB9↑,PSMA6↑,UBE4B↓,PSME2↑,BIRC6↓,UBE2L6↑,HLA-C↑,USP22↓,PSMA4↑,USP46↓,TAP2↑,HSPB1↓ |
| Dendritic Cell Maturation | 4.81E+00 | 1.14E-01 | 3.13 | B2M↑,FCGR2C↑,IL1A↑,ICAM1↑,MYD88↑,HLA-A↑,HLA-B↑, IFNB1↑,IKBKE↑, JAK2↑, IL33↑,TLR2↑,IL36G↑,PLCB4↓,PLCE1↓,HLA-C↑, IL1RN↑, STAT2↑, TLR3↑, STAT1↑, ATM↓ |
| Role of RIG1-like Receptors in Antiviral Innate Immunity | 4.47E+00 | 2.14E-01 | 1.667 | IFIH1↑,DHX58↑,IRF7↑,DDX58↑,IFNB1↑,MAVS↓,IKBKE↑,CASP8↑,CASP10↑ |
| Glycogen Degradation II | 4.42E+00 | 4.55E-01 | NaN | PGM1↓,PYGL↓,TYMP↑,AGL↓,MTAP↓ |
| Type I Diabetes Mellitus Signaling | 4.04E+00 | 1.31E-01 | 2.121 | MYD88↑,HLA-A↑,HLA-B↑,IKBKE↑,JAK2↑,IRF1↑,FAS↑,HLA-G↑,HLA-C↑,SOCS2↑,CASP8↑,HLA-F↑,STAT1↑,HLA-E↑ |
| Glycogen Degradation III | 4.01E+00 | 3.85E-01 | NaN | PGM1↓,PYGL↓,TYMP↑,AGL↓,MTAP↓ |
| Role of Hypercytokinemia/hyperchemokinemia in the Pathogenesis of Influenza | 3.73E+00 | 1.95E-01 | NaN | IL33↑,CXCL10↑,IL36G↑,IL1A↑,IL1RN↑,IFNB1↑,CCL5↑,IFNL1↑ |
| Crosstalk between Dendritic Cells and Natural Killer Cells | 3.67E+00 | 1.35E-01 | NaN | HLA-G↑,IL2RG↑,IL15RA↑,HLA-C↑,HLA-A↑,HLA-B↑,IFNB1↑,TNFSF10↑,TLR3↑,HLA-F↑,FAS↑,HLA-E↑ |
| Pathogenesis of Multiple Sclerosis | 3.57E+00 | 4.44E-01 | NaN | CXCL10↑,CXCL11↑,CCL5↑,CXCL9↑ |
| UVA-Induced MAPK Signaling | 3.55E+00 | 1.24E-01 | 0.577 | PARP10↑,ZC3HAV1↑,PARP8↑,PARP12↑,PARP9↑,PARP1↓,MTOR↓,PLCB4↓,PLCE1↓,STAT1↑,PARP14↑,ATM↓,PRKCA↓ |
| Granulocyte Adhesion and Diapedesis | 3.44E+00 | 1.02E-01 | NaN | IL1A↑,SELL↑,CXCL11↑,ICAM1↑,CCL20↑,MMP13↑,CCL22↑,CCL5↑,CXCL9↑,GLG1↓,IL33↑,CXCL10↑,CXCL16↑,IL36G↑,IL1RN↑,CX3CL1↑,HSPB1↓ |
| Altered T Cell and B Cell Signaling in Rheumatoid Arthritis | 3.35E+00 | 1.33E-01 | NaN | IL33↑,TLR2↑,IL36G↑,IL1A↑,HLA-A↑,IL1RN↑,TNFSF13↑,HLA-B↑,TLR3↑,FAS↑,TNFSF13B↑ |
| Valine Degradation I | 3.25E+00 | 2.78E-01 | NaN | HIBCH↓,HIBADH↓,DBT↓,ALDH6A1↓,BCKDHB↓ |
| Allograft Rejection Signaling | 3.24E+00 | 1.67E-01 | NaN | B2M↑,HLA-G↑,HLA-C↑,HLA-A↑,HLA-B↑,HLA-F↑,FAS↑,HLA-E↑ |
| LPS/IL-1 Mediated Inhibition of RXR Function | 3.15E+00 | 9.13E-02 | 2.449 | IL1A↑,CPT1A↓,SLC27A2↓,MYD88↑,GSTA4↓,ACOX1↓,IL33↑,HSTST3B1↓,IL36G↑,SULT1E1↓,UST↓,IL1RN↑,CAT↓,GSTA1↓,ALDH18A1↓,CYP2B6↓,ALDH6A1↓,ALDH7A1↓,PPARGC1A↓ |
| Agranulocyte Adhesion and Diapedesis | 3.15E+00 | 9.66E-02 | NaN | MYH10↓,IL1A↑,SELL↑,CXCL11↑,ICAM1↑,CCL20↑,MMP13↑,CCL22↑,CCL5↑,CXCL9↑,GLG1↓,IL33↑,CXCL10↑,CXCL16↑,IL36G↑,IL1RN↑,CX3CL1↑ |
| Apoptosis Signaling | 3.09E+00 | 1.24E-01 | 1.508 | BIRC6↓,IKBKE↑,CASP8↑,BCL2A1↑,BIRC3↑,BAK1↑,FAS↑,PRKCA↓,PARP1↓,CAPN3↑,CASP10↑ |
| Aryl Hydrocarbon Receptor Signaling | 2.99E+00 | 1.04E-01 | 1 | RBL2↓,IL1A↑,GSTA4↓,CDK4↓,FAS↑,TGM2↑,CCNA1↑,GSTA1↓,ALDH18A1↓,ATR↓,ALDH6A1↓,ALDH7A1↓,ATM↓,HSPB1↓ |
| Role of PKR in Interferon Induction and Antiviral Response | 2.97E+00 | 1.71E-01 | NaN | IFNB1↑,IKBKE↑,TLR3↑,EIF2AK2↑,CASP8↑,STAT1↑,IRF1↑ |
| Inflammasome pathway | 2.92E+00 | 2.38E-01 | 2.236 | AIM2↑,MYD88↑,CASP1↑,CASP8↑,CASP5↑ |
| Cytotoxic T Lymphocyte-mediated Apoptosis of Target Cells | 2.90E+00 | 1.94E-01 | NaN | B2M↑,HLA-C↑,HLA-A↑,HLA-B↑,CASP8↑,FAS↑ |
| Autoimmune Thyroid Disease Signaling | 2.90E+00 | 1.67E-01 | NaN | HLA-G↑,HLA-C↑,HLA-A↑,HLA-B↑,HLA-F↑,FAS↑,HLA-E↑ |
| NF-ÎºB Signaling | 2.67E+00 | 8.99E-02 | 1 | IL1A↑,TGFBR1↓,MYD88↑,BMPR1B↓,IGF2R↓,IL33↑,TLR2↑,IL36G↑,TNIP1↑,IL1RN↑,IGF1R↓,EIF2AK2↑,TLR3↑,CASP8↑,TNFSF13B↑,ATM↓ |
| Induction of Apoptosis by HIV1 | 2.59E+00 | 1.33E-01 | 0 | SLC25A6↓,IKBKE↑,IKBKAP↓,CASP8↑,SLC25A5↓,BIRC3↑,BAK1↑,FAS↑ |
| Toll-like Receptor Signaling | 2.53E+00 | 1.20E-01 | 3 | IL33↑,TLR2↑,IL36G↑,IL1A↑,MYD88↑,IL1RN↑,TLR3↑,EIF2AK2↑,IRAK2↑ |
| GDP-glucose Biosynthesis | 2.51E+00 | 3.75E-01 | NaN | HK1↓,HK2↑,PGM1↓ |
| Atherosclerosis Signaling | 2.39E+00 | 9.68E-02 | NaN | APOL1↑,IL33↑,ALOX15↓,IL36G↑,IL1A↑,ICAM1↑,IL1RN↑,MMP13↑,PCYOX1↓,PLA2G12A↓,APOD↑,GLG1↓ |
| Glucose and Glucose-1-phosphate Degradation | 2.35E+00 | 3.33E-01 | NaN | HK1↓,HK2↑,PGM1↓ |
| Glutathione Biosynthesis | 2.33E+00 | 6.67E-01 | NaN | GCLC↓,GCLM↓ |
| D-glucuronate Degradation I | 2.33E+00 | 6.67E-01 | NaN | AKR1A1↓,CRYL1↓ |
| Glycerol-3-phosphate Shuttle | 2.33E+00 | 6.67E-01 | NaN | GPD2↑,GPD1L↓ |
| Neuroprotective Role of THOP1 in Alzheimer's Disease | 2.29E+00 | 9.82E-02 | NaN | C1R↑,HLA-G↑,HLA-C↑,HLA-A↑,HLA-B↑,PRKAR2A↓,IDE↓,HLA-F↑,FAP↑,KLK10↑,HLA-E↑ |
| TREM1 Signaling | 2.18E+00 | 1.14E-01 | 2.828 | TLR2↑,NLRC5↑,ICAM1↑,MYD88↑,CASP1↑,JAK2↑,TLR3↑,CASP5↑ |
| OX40 Signaling Pathway | 2.18E+00 | 1.25E-01 | NaN | B2M↑,HLA-G↑,HLA-C↑,HLA-A↑,HLA-B↑,HLA-F↑,HLA-E↑ |
| AMPK Signaling | 2.14E+00 | 7.92E-02 | -1.155 | PFKFB3↓,CPT1A↓,SLC2A1↓,EEF2↓,PRKAR2A↓,PFKM↓,MTOR↓,PPP2R3A↓,PPM1L↓,PRKAA2↓,ACACA↓,SMARCC1↓,HLTF↓,PHF10↓,ATM↓,PPARGC1A↓ |
| iNOS Signaling | 2.11E+00 | 1.36E-01 | 2.236 | MYD88↑,IKBKE↑,JAK2↑,STAT1↑,IRF1↑,IRAK2↑ |
| Acute Phase Response Signaling | 2.10E+00 | 8.28E-02 | 1.508 | IL1A↑,SERPING1↑,MYD88↑,C1S↑,IKBKE↑,JAK2↑,IL33↑,C1R↑,MTOR↓,FTL↓,IL36G↑,IL1RN↑,CFB↑,SOCS2↑ |
| UDP-N-acetyl-D-galactosamine Biosynthesis II | 2.08E+00 | 2.73E-01 | NaN | HK1↓,GPI↓,HK2↑ |
| Regulation of eIF4 and p70S6K Signaling | 2.05E+00 | 8.44E-02 | 0 | PABPC1↓,EIF4EBP2↓,RPS23↓,EIF3E↓,EIF3F↓,MTOR↓,RPS16↓,PPP2R3A↓,PPM1L↓,RPS5↓,EIF3L↓,ATM↓,EIF3K↓ |
| Branched-chain α-keto acid Dehydrogenase Complex | 2.04E+00 | 5.00E-01 | NaN | DBT↓,BCKDHB↓ |
| Phagosome Maturation | 2.03E+00 | 8.70E-02 | NaN | B2M↑,DYNC1H1↓,ATP6V1C2↓,HLA-C↑,HLA-A↑,CTSS↑,HLA-B↑,DYNC2H1↓,DYNLT1↑,TAP1↑,ATP6V1B1↓,PRDX2↓ |
| NRF2-mediated Oxidative Stress Response | 2.03E+00 | 7.89E-02 | -2.828 | AKR7A2↓,GSTA4↓,GCLC↓,DNAJC10↓,DNAJA1↑,FTL↓,AKR1A1↓,DNAJC4↓,CAT↓,GSTA1↓,GCLM↓, ATM↓,PRKCA↓,EPHX1↓ |
| Role of Macrophages, Fibroblasts and Endothelial Cells in Rheumatoid Arthritis | 2.00E+00 | 6.95E-02 | NaN | IL1A↑,ICAM1↑,MYD88↑,LRP6↓,VEGFC↑,MMP13↑,IKBKE↑,CCL5↑,JAK2↑,IL33↑,TLR2↑,IL36G↑,PLCB4↓,PLCE1↓,IL1RN↑,TLR3↑,LRP1↓,TNFSF13B↑,IRAK2↑,ATM↓,PRKCA↓ |
| Production of Nitric OxIDE and Reactive Oxygen Species in Macrophages | 1.97E+00 | 7.77E-02 | 2.324 | PPP1R3C↓,IKBKE↑,JAK2↑,PCYOX1↓,IRF1↑,APOL1↑,TLR2↑,RND3↑,PPP2R3A↓,PPM1L↓,CAT↓,STAT1↑,APOD↑,ATM↓,PRKCA↓ |
| EIF2 Signaling | 1.95E+00 | 7.55E-02 | -2.121 | PABPC1↓,RPS23↓,RPL26↓,RPL12↓,EIF3E↓,EIF3F↓,WARS↑,RPS16↓,IGF1R↓,RPL6↓,EIF2AK2↑,RPS5↓,EIF3L↓,EIF3K↓,ATM↓,RPLP0↓ |
| Tryptophan Degradation III (Eukaryotic) | 1.91E+00 | 1.74E-01 | NaN | ACAT1↓,IDO1↑,KYNU↑,HADH↓ |
| Role of BRCA1 in DNA Damage Response | 1.90E+00 | 1.03E-01 | -1 | RBL2↓,ATR↓,SMARCC1↓,STAT1↑,BRIP1↑,HLTF↓,PHF10↓,ATM↓ |
| MTOR Signaling | 1.89E+00 | 7.61E-02 | 0.378 | RPS23↓,VEGFC↑,EIF3E↓,EIF3F↓,MTOR↓,RPS16↓,RND3↑,PPP2R3A↓,PPM1L↓,PRKAA2↓,RPS5↓,EIF3L↓,ATM↓,PRKCA↓,EIF3K↓ |
| NAD biosynthesis II (from tryptophan) | 1.87E+00 | 2.31E-01 | NaN | NADSYN1↓,IDO1↑,KYNU↑ |
| Tumoricidal Function of Hepatic Natural Killer Cells | 1.85E+00 | 1.67E-01 | NaN | SERPINB9↑,ICAM1↑,CASP8↑,FAS↑ |
| Role of JAK1, JAK2 and TYK2 in Interferon Signaling | 1.85E+00 | 1.67E-01 | NaN | IFNB1↑,STAT2↑,JAK2↑,STAT1↑ |
| Trehalose Degradation II (Trehalase) | 1.83E+00 | 4.00E-01 | NaN | HK1↓,HK2↑ |
| IL-17A Signaling in Gastric Cells | 1.79E+00 | 1.60E-01 | NaN | CXCL10↑,CXCL11↑,CCL20↑,CCL5↑ |
| DNA Double-Strand Break Repair by Non-Homologous End Joining | 1.78E+00 | 2.14E-01 | NaN | PRKDC↓,ATM↓,PARP1↓ |
| Aldosterone Signaling in Epithelial Cells | 1.78E+00 | 7.78E-02 | -2.828 | SLC12A2↓,DNAJC10↓,DNAJA1↑,PLCB4↓,PLCE1↓,DNAJC4↓,NR3C2↓,SCNN1B↓,PI4KA↓,ATM↓,HSPA4L↓,HSPB1↓,PRKCA↓ |
| Role of Cytokines in Mediating Communication between Immune Cells | 1.77E+00 | 1.15E-01 | NaN | IL33↑,IL36G↑,IL1A↑,IL1RN↑,IFNB1↑,IFNL1↑ |
| Antiproliferative Role of TOB in T Cell Signaling | 1.73E+00 | 1.54E-01 | NaN | PABPC1↓,TGFBR1↓,CCNA1↑,PABPC4↓ |
| p38 MAPK Signaling | 1.68E+00 | 8.40E-02 | 1.265 | IL33↑,IL36G↑,IL1A↑,TGFBR1↓,IL1RN↑,STAT1↑,FAS↑,IRAK2↑,PLA2G12A↓,HSPB1↓ |
| LXR/RXR Activation | 1.64E+00 | 8.26E-02 | -1.414 | APOL1↑,IL33↑,IL36G↑,IL1A↑,IL1RN↑,ACACA↓,PCYOX1↓,TLR3↑,HADH↓,APOD↑ |
| Xenobiotic Metabolism Signaling | 1.62E+00 | 6.67E-02 | NaN | IL1A↑,GSTA4↓,GCLC↓,HSTST3B1↓,FTL↓,SULT1E1↓,UST↓,PPP2R3A↓,CAT↓,PPM1L↓,GSTA1↓,ALDH18A1↓,CYP2B6↓,ALDH6A1↓,PRKCA↓,ALDH7A1↓,ATM↓,PPARGC1A↓ |
| Granzyme B Signaling | 1.62E+00 | 1.88E-01 | NaN | PRKDC↓,CASP8↑,PARP1↓ |
| Î³-glutamyl Cycle | 1.62E+00 | 1.88E-01 | NaN | CHAC1↑,GCLC↓,GCLM↓ |
| IL-15 Production | 1.62E+00 | 1.43E-01 | NaN | IFNB1↑,JAK2↑,STAT1↑,IRF1↑ |
| Systemic Lupus Erythematosus Signaling | 1.55E+00 | 6.91E-02 | NaN | FCGR2C↑,IL1A↑,HLA-A↑,PRPF8↓,HLA-B↑,IL33↑,HLA-G↑,IL36G↑,MTOR↓,IL1RN↑,HLA-C↑,HLA-F↑,TNFSF13B↑,HLA-E↑,ATM↓ |
| Inositol Pyrophosphates Biosynthesis | 1.53E+00 | 2.86E-01 | NaN | PPIP5K1↓,PPIP5K2↓ |
| Tryptophan Degradation to 2-amino-3-carboxymuconate Semialdehyde | 1.53E+00 | 2.86E-01 | NaN | IDO1↑,KYNU↑ |
| IL-12 Signaling and Production in Macrophages | 1.53E+00 | 7.64E-02 | NaN | APOL1↑,TLR2↑,ALOX15↓,MYD88↑,IKBKE↑,PCYOX1↓,STAT↑1,IRF1↑,ATM↓,APOD↑,PRKCA↓ |
| Huntington's Disease Signaling | 1.50E+00 | 6.67E-02 | -3.317 | HDAC9↑,PSME2↑,CASP4↑,POLR2B↓,TGM2↑,GNB4↑,MTOR↓,PLCB4↓,CASP1↑,IGF1R↓,CASP8↑,CASP5↑,CASP10↑,ATM↓,CAPN3↑,PRKCA↓ |
| p53 Signaling | 1.47E+00 | 8.11E-02 | 1.134 | PRKDC↓,HDAC9↑,PMAIP1↑,CDK4↓,ATR↓,PML↑,TNFRSF10A↑,FAS↑,ATM↓ |
| Cyclins and Cell Cycle Regulation | 1.47E+00 | 9.09E-02 | 1.633 | HDAC9↑,CCNA1↑,PPP2R3A↓,PPM1L↓,CDK4↓,ATR↓,ATM↓ |
| Osteoarthritis Pathway | 1.44E+00 | 6.83E-02 | 2.673 | TGFBR1↓,VEGFC↑,MMP13↑,CASP4↑,TLR2↑,MTOR↓,CASP1↑,PRKAA2↓,SOX9↑,CASP8↑,CASP5↑,LRP1↓,CASP10↑,PPARGC1A↓ |
| Role of JAK2 in Hormone-like Cytokine Signaling | 1.43E+00 | 1.25E-01 | NaN | SOCS2↑,JAK2↑,STAT1↑,HLTF↓ |
| Ethanol Degradation II | 1.43E+00 | 1.25E-01 | NaN | AKR1A1↓,ADH1C↓,ADH1B↓,ALDH7A1↓ |
| Salvage Pathways of Pyrimidine DeoxyribonucleotIDEs | 1.42E+00 | 2.50E-01 | NaN | TYMP↑,APOBEC3G↑ |
| GADD45 Signaling | 1.42E+00 | 1.58E-01 | NaN | CDK4↓,ATR↓,ATM↓ |
| Methylthiopropionate Biosynthesis | 1.40E+00 | 1.00E+00 | NaN | ADI1↓ |
| S-methyl-5'-thioadenosine Degradation II | 1.40E+00 | 1.00E+00 | NaN | MTAP↓ |
| CTLA4 Signaling in Cytotoxic T Lymphocytes | 1.37E+00 | 8.16E-02 | NaN | B2M↑,PPP2R3A↓,HLA-C↑,HLA-A↑,HLA-B↑,PPM1L↓,JAK2↑,ATM↓ |
| Phagosome Formation | 1.37E+00 | 7.76E-02 | NaN | TLR2↑,FCGR2C↑,CLEC7A↑,PLCB4↓,PLCE1↓,RND3↑,TLR3↑,ATM↓,PRKCA↓ |
| Prolactin Signaling | 1.32E+00 | 8.43E-02 | 0 | SOCS2↑,NMI,JAK2↑,STAT1↑,IRF1↑,ATM↓,PRKCA↓ |
| JAK/Stat Signaling | 1.32E+00 | 8.43E-02 | 0.378 | MTOR↓,PIAS2↓,SOCS2↑,STAT2↑,JAK2↑,STAT1↑,ATM↓ |
